# Characterization of Two Wheat-*Thinopyrum ponticum* Introgression Lines With Pyramiding Resistance to Powdery Mildew

**DOI:** 10.3389/fpls.2022.943669

**Published:** 2022-07-15

**Authors:** Mingzhu Li, Yuanyuan Yuan, Fei Ni, Xingfeng Li, Honggang Wang, Yinguang Bao

**Affiliations:** ^1^State Key Laboratory of Crop Biology, Agronomy College of Shandong Agricultural University, Tai'an, China; ^2^Bureau of Agriculture and Rural Affairs of Linqing, Liaocheng, China; ^3^Crop Research Institute, Jinan Academy of Agricultural Sciences, Jinan, China

**Keywords:** wheat, powdery mildew, *Thinopyrum ponticum*, introgression, GISH, FISH

## Abstract

Powdery mildew is one of the most devastating foliar diseases in wheat production. The wild relative *Thinopyrum ponticum* (2*n* = 10*x* = 70) has been widely used in wheat genetic improvement due to its superior resistance to both biotic and abiotic stresses. In the present study, two wheat-*Th. ponticum* introgression lines named SN0293-2 and SN0293-7 were developed from the progenies of a cross between the octoploid *Trititrigia* SNTE20 and common wheat, including the elite cultivar Jimai 22. They had a novel powdery mildew resistance gene (temporarily named *PmSN0293*) putatively from *Th. ponticum* pyramided with *Pm2* and *Pm52*, exhibiting excellent *Pm* resistance at both the seedling and adult stages. Sequential GISH-FISH detected no signal of *Th. ponticum* in these two lines but a pair of T1BL·1RS in SN0293-2. Chromosomal structural variations were also observed obviously in SN0293-2 and SN0293-7. Through the Wheat 660K SNP array, 157 SNPs, 134 of which were on 6A, were found to be specific to *Th. ponticum*. Based on the data combined with DNA re-sequencing, seven specific markers, including one CAPS marker on 2B and six CAPS and Indel markers on 6A, were developed, confirming their wheat-*Th. ponticum* introgression nature. Furthermore, the two lines displayed positive plant height and produced more kernels and higher 1,000-grain weight. Excellent resistance with desirable agronomic traits makes them valuable in wheat breeding programs.

## Introduction

As one of the most important staple food crops, wheat provides more than 20% of the calories and the protein for the world's population (Braun et al., 2010). The need for the production of wheat is continuously growing because of the increasing population (Hawkesford et al., 2013). However, it is threatened by many diseases which reduce yield and decrease quality.

Powdery mildew, caused by *Blumeria graminis* f. sp. *tritici* (*Bgt*), is one of the most devastating wheat diseases across the world. It can affect wheat photosynthesis and consequently decrease plant growth and grain filling, resulting in yield reductions (Zhang et al., 2020). Although fungicides can effectively control this disease, they also cause environmental pollution and cost increases. Host resistance is considered to be the most economical and environment-friendly means to do so (Wang et al., 2005; Liu et al., 2017). Therefore, it is a key step to explore and utilize resistance genes in wheat breeding programs. To date, more than 100 designated powdery mildew (*Pm*) resistance genes/alleles in 63 loci (*Pm*1-*Pm68*) have been documented (He et al., 2021; Gao et al., 2022). Some of them have been cloned, such as the broad-spectrum resistance gene *Pm21* from *Dasypyrum villosum* (2*n*= 2x = 14, VV) (He et al., 2018; Xing et al., 2018). However, with the rapid evolution of new *Bgt* isolates, the single resistance gene is easily overcome. For instance, the well-known *Pm8* on rye (*Secale cereale* L., 2*n* = 2x = 14, RR) chromosome arm 1RS has lost its function against new *Bgt* isolates, such as E09 (Ren et al., 2017). Hence, it is of great necessity to explore new *Pm* genes and pyramid multiple ones to broaden the resistance spectrum.

Wild relatives of common wheat carry many valuable genes that can be used for wheat improvement. The tall wheatgrass *Thinopyrum ponticum* (Podp.) Barkworth & D. R. Dewey (2*n* =10*x* =70, StStStStE^e^E^e^E^b^E^b^E^x^E^x^ or JJJJJJJ^S^J^S^J^S^J^S^) has long been known to have superior resistance to both biotic and abiotic stresses, including powdery mildew, stem rust, leaf rust, stripe rust, eyespot and Fusarium head blight (Li and Wang, 2009). Because of its ability to readily be crossed with wheat, many genes for disease resistance have been introduced into wheat. Among them, eleven were formally documented, including *Lr19, Lr24, Lr29, Sr24, Sr25, S26, Sr43, Yr69, Cmc2, Fhb7*, and *Pm51* (Sarma and Knott, 1966; Hart et al., 1976; Whelan and Lukow, 1990; Procunier et al., 1995; Mago et al., 2005; Li and Wang, 2009; Niu et al., 2014; Zhan et al., 2014; Hou et al., 2016; Wang et al., 2020). *Pm51* is the only *Pm* gene designated officially from *Th. ponticum* so far. Due to the allodecaploid nature, *Th. ponticum* has a large and complex genome, and resistance to a disease might be associated with its different chromosomes (Chen et al., 1998; Li and Wang, 2009). Therefore, there might be novel *Pm* gene(s) to be explored in *Th. ponticum*.

SNTE20, a wheat-*Th. ponticum* partial amphiploid with powdery mildew resistance, was previously developed in our lab (He et al., 2013). In the present study, crosses between SNTE20 and common wheat were carried out, and consequently two *Trititrigia* introgression lines were generated, SN0293-2 and SN0293-7, both of which were resistant to powdery mildew. The two introgression lines were then characterized by combined methods of morphology, disease evaluation, sequential genomic *in situ* hybridization-fluorescence *in situ* hybridization (GISH-FISH), and molecular marker analyses.

## Materials and Methods

### Plant Materials

Materials used in this study included *Th. ponticum*, rye, SNTE20, Yannong15 (YN15), Shannongfu63 (SNF63), Jimai22 (JM22), Shannong224 (SN224), SN0293-2, SN0293-7, CH7086, and Huixianhong (HXH). *Th. ponticum* (accession No. R431) was provided by Prof. Zhensheng Li, the former Northwest Institute of Botany, the Chinese Academy of Sciences, Yangling, China. Wheat-*Th. ponticum* octoploid SNTE20 was developed from the multiple cross *Th. ponticum*/YN15//SNF63. YN15, SNF63, and JM22 are wheat cultivars. SN224 is a T1BL·1RS translocation line with dwarf stems. SN0293-2 and SN0293-7 were generated from a hybrid of the cross SNTE20/YN15//SN224/3/JM22 (Supplementary Figure 1). CH7086 carrying *Pm51* was provided by Prof. Xiaojun Zhang, the former Crop Science Institute, Shanxi Academy of Agricultural Sciences, Taiyuan, China.

### Assessment of Agronomic Traits

Fifteen plants were grown in each 1.5 m long row, with 25 cm spacing between the rows. Agronomic traits, including plant height, spike length, spikelets per spike, kernels per spike, and thousand kernels weight, were recorded at the Experimental Station of Shandong Agricultural University. Each trait was averaged on 10 plants.

### Evaluation of Powdery Mildew Resistance

Seedling resistance assessment was performed in a growth chamber using the *Bgt* isolate E09, following the method described by Zhao et al. (2013). Seedlings were grown in rectangular plastic trays (5 cm × 5 cm; 10 plants per tray) and inoculated with fresh *Bgt* conidiospores obtained from the susceptible cultivar HXH at the one-leaf stage. After approximately 2 weeks, when symptoms were severe on HXH, infection types (ITs) on the plants were described using a 0–4 infection scale: 0–2 scores indicating resistance and 3–4 susceptibility.

At the adult stage, resistance to powdery mildew was evaluated after natural infection in field-grown plants at the Experimental Station of Shandong Agricultural University over three growing seasons (2018–2020). The most severe reaction type in a given year was considered to be the final resistance result. HXH was planted perpendicular and adjacent to the test rows to serve as an inoculum spreader and a susceptible control. The disease symptoms were recorded three times at weekly intervals after flowering, and the most severe infection score was used as the final response. The ITs of powdery mildew at the adult stage were scored using a 0–9 scale: 0–4 scores indicating resistance and 5–9 susceptibility (Li et al., 2011).

### Identification of Sequential GISH-FISH

The chromosomes were prepared following the method described by Kato et al. (2004). The purified total genomic DNA extracted from *Th. ponticum* or rye was labeled with Texas Red-5-dCTP probes, with the sheared genomic DNA from YN15 as a blocker. GISH analysis was performed as described by Fu et al. (2012). For FISH analysis, oligonucleotide probes, including TAMRA (6-carboxytetramethylrhodamine)-labeled oligonucleotides pAs1-1, pAs1-3, pAs1-4, pAs1-6, AFA-3, and AFA-4 and FAM (6-carboxyfluorescein)-labeled oligonucleotides pSc119.2-1 and (GAA)_10_, were used. All probes were synthesized by Sangon Biotech Co., Ltd. (Shanghai, China). FISH analysis was performed as described by Huang et al. (2018). The chromosomes were counterstained with 4, 6-diamidino-2-phenylindole (DAPI), and the images were captured with a fluorescence microscope (Olympus BX60) equipped with a CCD (charge-coupled device) camera.

### Development of Molecular Markers and PCR Amplification

The Axiom Wheat 660K Genotyping Array was used to genotype SN0293-2 and their parents YN15, SNF63, JM22, SN224, SNTE20 and *Th. ponticum*. The SNP typing data were processed using Microsoft Excel 2016 software, regarding SNPs present in SN0293-2, SNTE20 and *Th. ponticum* and absent in the comment wheat parents as the putatively specific ones of *Th. ponticum*. Library construction and high-throughput sequencing of SN0293-2, SNF63, and SNTE20 were performed by Berry Genomics Company (Beijing, China). The raw reads were subjected to a quality check and then filtered by fastp to remove adapter sequences and low-quality bases (Chen et al., 2018). High-quality reads were mapped to the *Triticeae* repeat database mipsREdat 9.3p (PGSB Repeat Database) using Burrows-Wheeler Aligner in order to filter out repeat noises, non-mapped reads (from the genome region of non-repeat sequences) were fished by homemade scripts and then used for variant calling (Li and Durbin, 2010). GATK 3.8 (https://www.software.broadinstitute.org/gatk) was used to call out all the variations, including SNPs and InDels. Markers of cleaved amplified polymorphic sequences (CAPS) were designed on the basis of SNPs, and the corresponding restriction endonucleases were used for restriction digestion. InDel markers were designed according to the results of resequencing. Briefly, the corresponding 500-bp both upstream and downstream sequences of the InDels larger than 10 bp were fished from the Chinese Spring reference on the website WheatOmics (Ma et al., 2021). About 1-kb fragments corresponding the InDels were used to perform local BLAST to identify genome specific fragments for marker development (identity > 95%, number of hits ≤ 3). The conserved sequences flanking the target region were then used to design primers.

DNA was isolated from young leaf tissues following a standard CTAB method. The PCR cycling condition was as follows: 95°C for 3 min, followed by 35 cycles of 95°C for 30 s, appropriate anneal temperature (50–60°C) for 40 s, 72°C for 1 min, and a final extension at 72°C for 5 min, and the amplification products were kept at 10°C using a Bio-Rad 9600 Thermal Cycler (Hercules, USA). PCR products amplified by CAPS markers were separated through 1% agarose electrophoresis and PCR products amplified by Indel markers were separated on 8% non-denatured polyacrylamide gels (39 acrylamide: 1 bisacrylamide).

Restriction endonuclease (*HaeIII, SacII, BstEII*) were selected for the CAPS marker and the digestion reaction was carried out according to the manufacturer's instructions (New England Biolabs, England). The digestion was conducted in a 10 μL reaction volume, contained 50 ng PCR products and digested at 37°C or 65°C (depending on the enzyme). The digestion reaction products were separated by 2% agarose electrophoresis.

## Results

### Evaluation of Powdery Mildew Resistance

**Figure 1:**
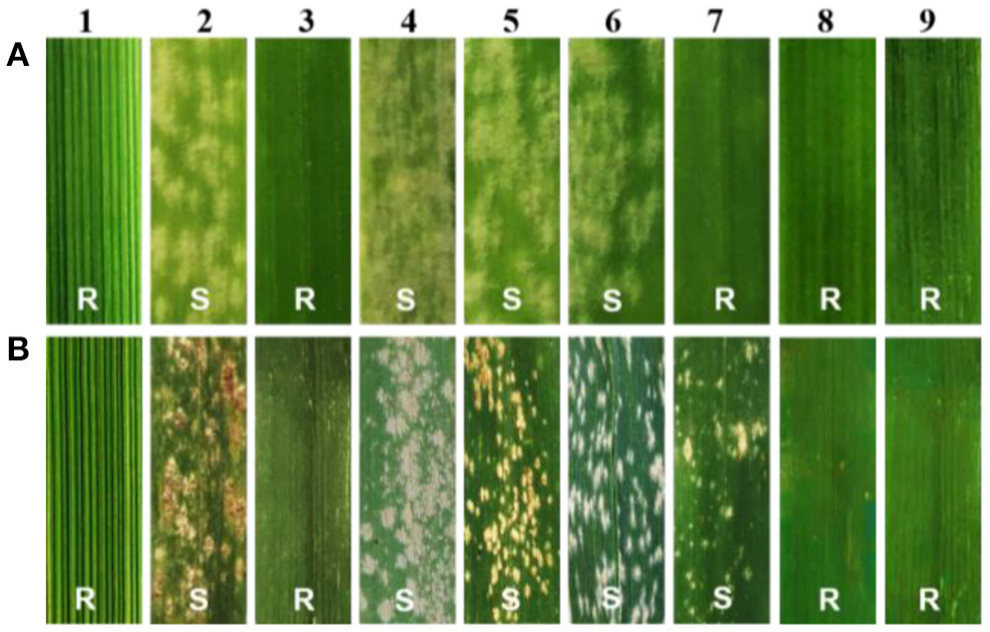
Evaluation of powdery mildew resistance. **(A)** The seedling stage. **(B)** The adult stage. 1–9 refer to *Th. ponticum*, HXH, SNTE20, SNF63, YN15, SN224, JM22, SN0293-2, and SN0293-7, respectively.

At the seedling stage, SN0293-2, SN0293-7, and its parents were inoculated with the *Bgt* isolate E09, and the disease reaction was assessed once the control HXH displayed thoroughly susceptible symptoms. *Th. ponticum*, SNTE20, JM22, SN0293-2, and SN0293-7 were all immune to E09 (IT = 0), while the common wheat parents YN15, SNF63, and SN224 appeared to be susceptible with an IT score of 4 (Figure 1A), suggesting that the resistance to powdery mildew at the seeding stage in SN0293-2 and SN0293-7 originated from either *Th. ponticum* or JM22. The seedling reactions of SN0293-2, SN0293-7, and 42 wheat genotypes carrying documented *Pm* genes and gene combinations to 25 *Bgt* isolates were simultaneously recorded (Supplementary Table 1). The susceptible cultivar Chancellor exhibited a high IT (4). SN0293-2 and SN0293-7 were resistant to 23 isolates, indicating their broad-spectrum resistance. Additionally, *Pm51*, the only documented powdery mildew resistance gene putatively derived from *Th. ponticum*, was nearly immune to the isolate E20 (Zhan et al., 2014), but SN0293-2 and SN0293-7 were susceptible to E20, indicating that the resistance genes in SN0293-2 and SN0293-7 were different from *Pm51*.

**Figure 2:**
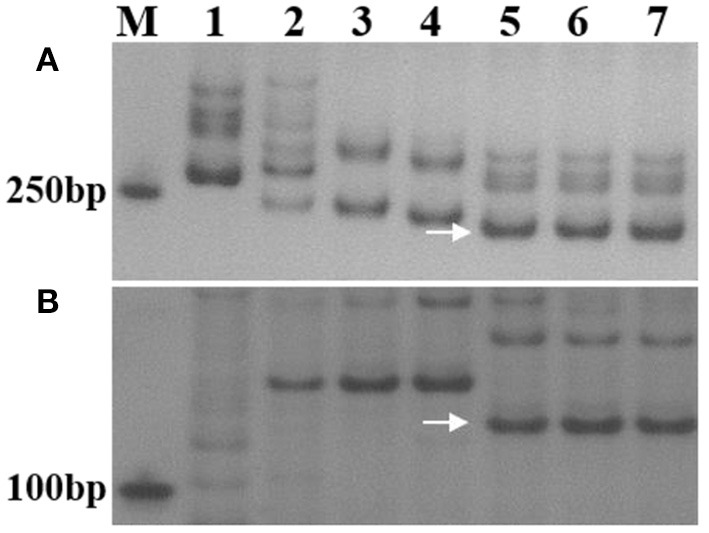
Amplified results of specific markers *Xcfd-81* and *Xicssl326*. **(A)** *Xcfd-81*. **(B)** *Xicssl326*. Lanes: M, DL2000 marker; 1–7 refer to *Th. ponticum*, SNTE20, YN15, SN224, JM22, SN0293-2, and SN0293-7, respectively. Arrows indicate specific bands.

At the adult stage, resistance to powdery mildew was tested in field over three growing seasons (2018–2020), and the most severe reaction type observed in a given year was considered to be the final result. It was found that SN0293-2, SN0293-7, *Th. ponticum*, and SNTE20 were resistant to powdery mildew, whereas YN15, SNF63, SN224, JM22, and CH7086 showed susceptibility (Figure 1B and Supplementary Figure 2). Thus, the resistance at the adult stage in SN0293-2 and SN0293-7, temporarily designated *PmSN0293*, was putatively inherited from *Th. ponticum* and different from *Pm51*. Previous studies have found that JM22 contains powdery mildew resistance genes *Pm2* and *Pm52* (Cao et al., 2010; Qu et al., 2020). To determine whether SN0293-2 and SN0293-7 had these two genes or not, we amplified the genomes of them and their parents by using the markers *Xcfd81* and *Xicssl326*, which were known to be linked with *Pm2* and *Pm52*, respectively. It was showed that both SN0293-2 and SN0293-7 contained the two genes derived from JM22 (Figure 2). The results above indicated that SN0293-2 and SN0293-7 might carry a new powdery mildew resistance gene *PmSN0293* from *Th. ponticum* pyramided with *Pm2* and *Pm52*.

### Cytogenetic Analyses

**Figure 3:**
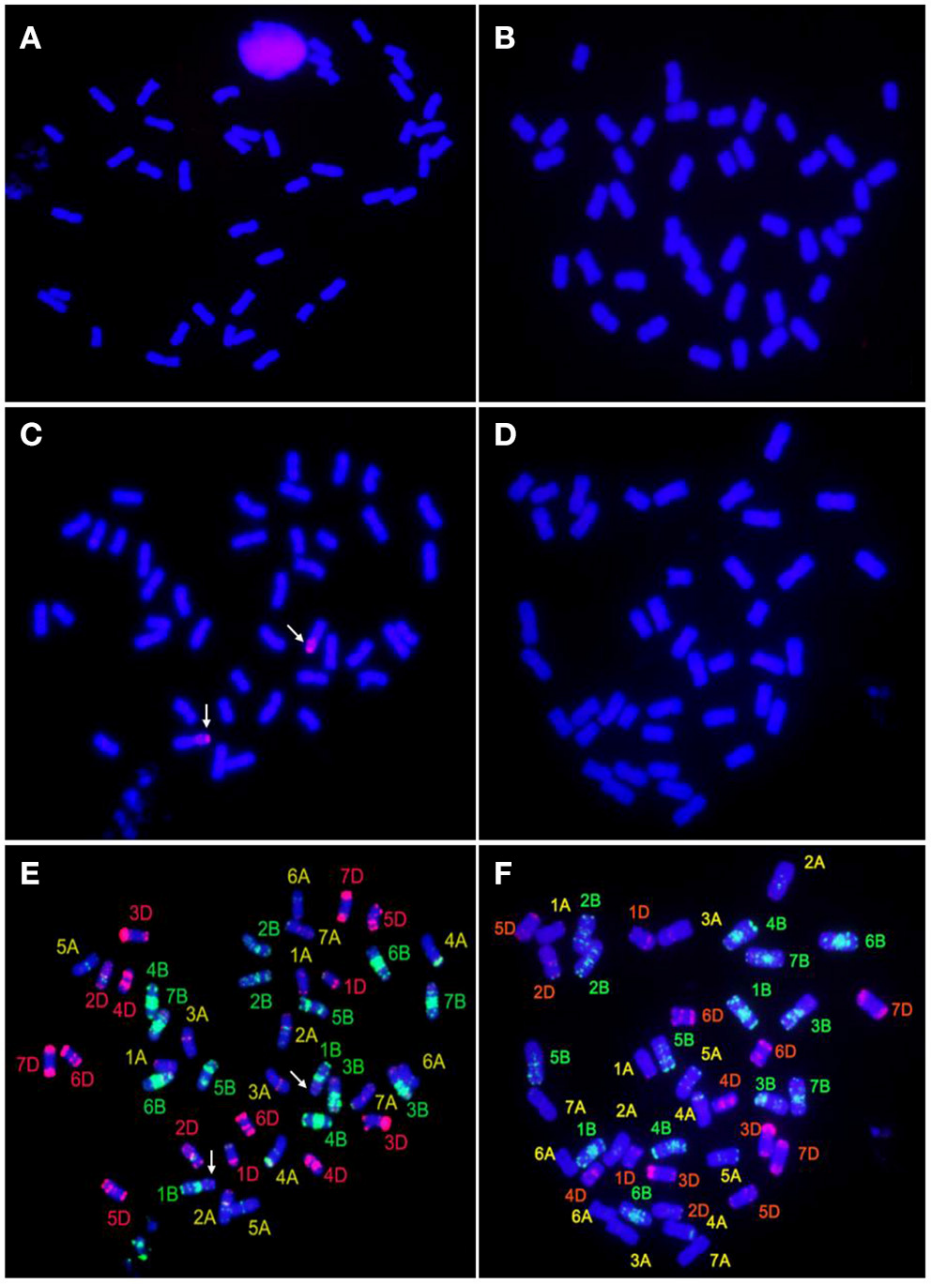
GISH-FISH analyses of SN0293-2 and SN0293-7. **(A)** GISH patterns of SN0293-2 probed with total genomic DNA of *Thinopyrum ponticum*. **(B)** GISH patterns of SN0293-7 probed with total genomic DNA of *Th. ponticum*. **(C)** GISH patterns of SN0293-2 probed with total genomic DNA of rye. **(D)** GISH patterns of SN0293-7 probed with total genomic DNA of rye. **(E)** FISH patterns of SN0293-2. **(F)** FISH patterns of SN0293-7. Arrows indicate the translocation T1BL·1RS.

GISH, probed with the total genomic DNA of *Th. ponticum* and blocked with the genomic DNA of YN15, revealed that both SN0293-2 and SN0293-7 had 42 chromosomes and no alien signal was detected (Figures 3A,B). Because the parent SN224 is a T1BL·1RS translocation line, the rye genomic DNA was further used as a probe. Two chromosome arms with red coloration alongside the blue chromosome arms of wheat were found in SN0293-2 and no hybridization signal in SN0293-7, indicating the rye chromosome arm 1RS was present in SN0293-2 and absent in SN0293-7 (Figures 3C,D).

**Figure 4:**
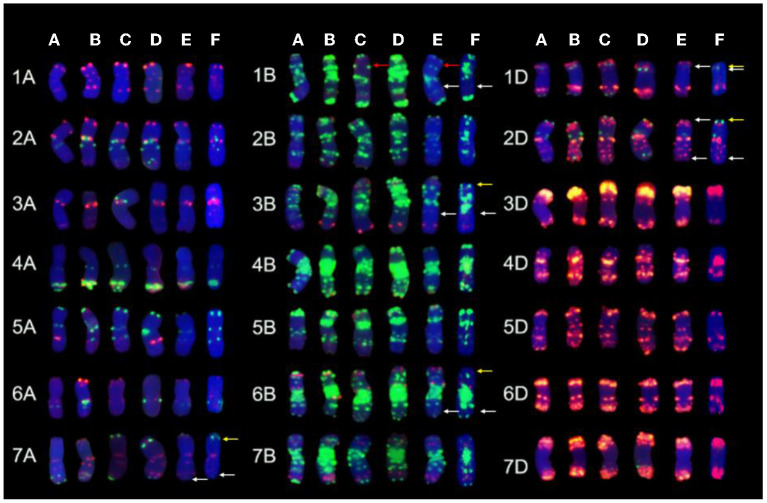
Chromosome comparison of FISH patterns among SN0293-2, SN0293-7, and their parents. **(A)** SNTE20. **(B)** YN15. **(C)** SN224. **(D)** JM22. **(E)** SN0293-2. **(F)** SN0293-7. The red, white, and yellow arrows indicate 1RS, FISH differences of SN0293-2 and SN0293-7, respectively.

After removing the GISH signals, the same slides were subjected to FISH analysis. The 1RSs in SN0293-2 were found in the form of T1BL·1RS, inheriting from the parent SN224 (Figure 3E). Further, FISH patterns of SN0293-2 and SN0293-7 in Figures 3E,F were compared with those of their parents (Figure 4). Differences between SN0293-2 and its parents were detected in the terminal region of 1DS, 2DS, 2DL, 6BL, and 7AL, and the middle of 3BL. As for SN0293-7, the terminal of 1DS, 2DL, 6BS, and 7AL as well as the middle of 1BL and 3BL appeared to be different from its parents. This suggested that chromosomes underwent structural variations with the formation of SN0293-2 and SN0293-7. In addition, differences between SN0293-2 and SN0293-7 were also present apart from the chromosome T1BL·1RS. For instance, red signals existed in the terminal regions of 1DS, 6BS, and 7AS in SN0293-2, but they disappeared in the corresponding regions of SN0293-7. The green signals in the telomere of SN0293-7 differed from those of SN0293-2.

### Development of Molecular Markers

**Figure 5:**
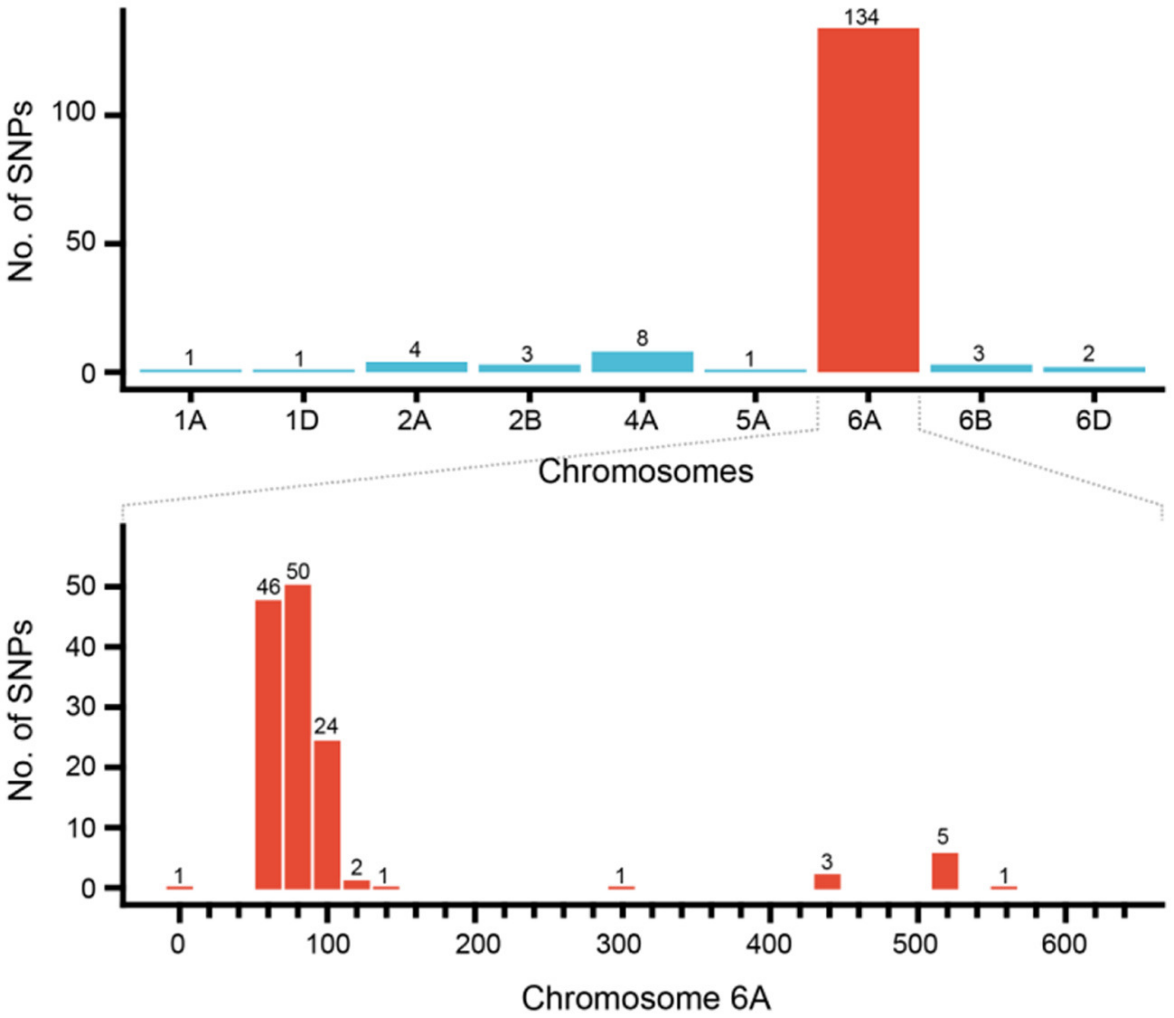
Distribution of SNPs specific to *Thinopyrum ponticum* in SN0293-2.

**Figure 6:**
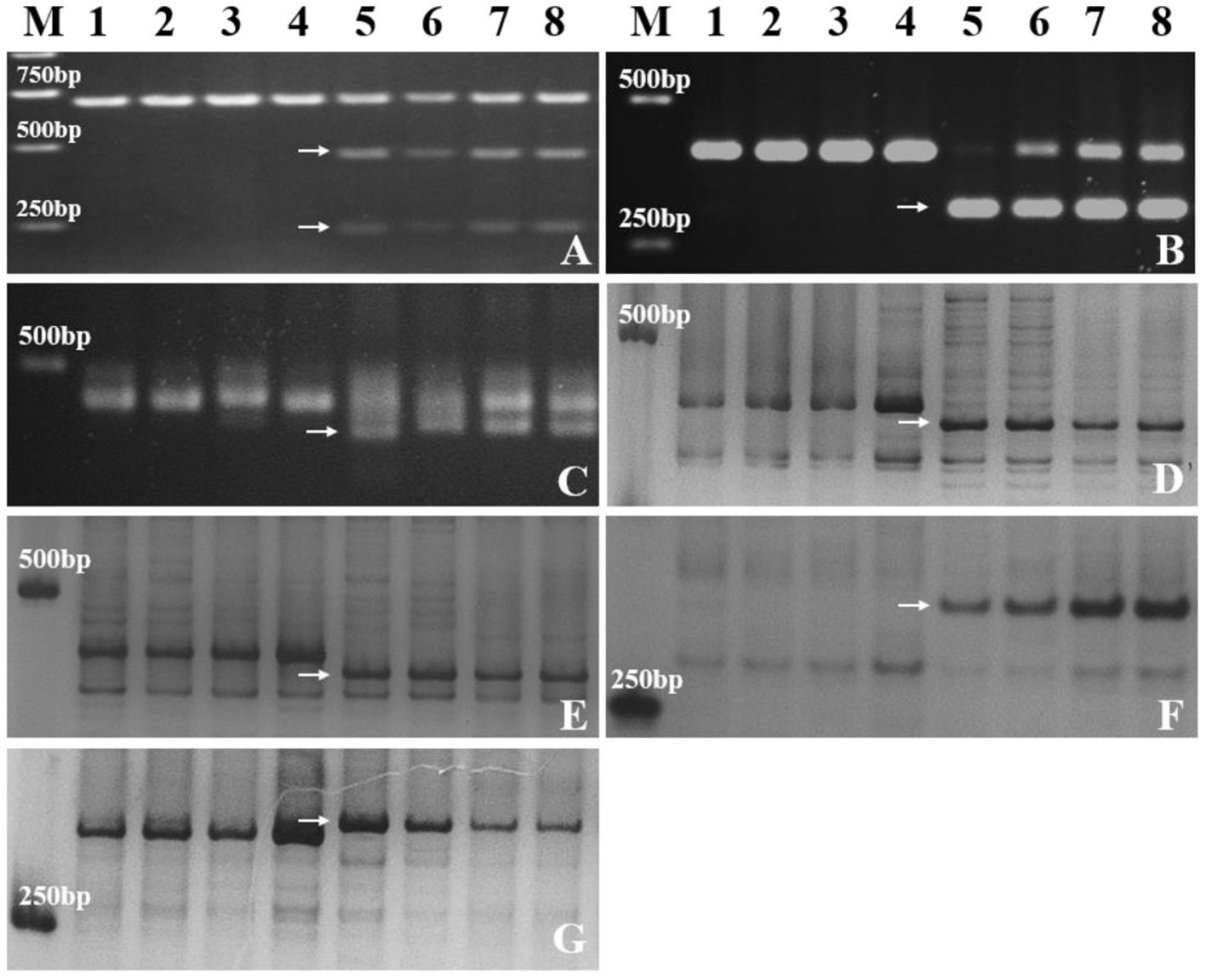
Molecular markers analysis of SN0293-2, SN0293-7 and their parents. **(A)** *CAPS421-HaeIII*. **(B)** *CAPS761-SacI*. **(C)** *CAPS564-BstEII*. **(D)** *Indel192*. **(E)** *Indel101*. **(F)** *Indel752*. **(G)** *Indel753*. Lanes: M, DL2000 marker; 1–8 refer to SNF63, YN15, SN224, JM22, *Th. ponticum*, SNTE20, SN0293-2, and SN0293-7, respectively. Arrows indicate specific bands of *Th. ponticum*.

A total of 157 specific SNP loci of *Th. ponticum* were obtained by using wheat 660K SNP array to analyze *Th. ponticum* and its parents, involving 9 chromosomes of three wheat subgenomes, of which 134 were located on chromosome 6A and mainly distributed in the physical interval of 60–110 Mb (Figure 5). DNA resequencing data showed that a total of 111 Indels larger than 10 bp were identified in the above 50 Mb region of chromosome 6A. These Indels were identical between the two lines SN0293 and SNTE20 containing *Th. ponticum* fragments, but different from the common wheat line SNF63. According to the results of Wheat 660K SNP array and resequencing data, three CAPS markers (*CAPS421-HaeIII, CAPS761-SacI, CAPS564-BstEII*) and four Indel markers (*Indel192, Indel101, Indel752, Indel753*) were obtained (Table 1). *Th. ponticum* specific bands were amplified in SN0293-2 and SN0293-7 (Figure 6), in which *CAPS421-HaeIII* was located on wheat chromosome 2B, *CAPS761-SacI, CAPS564-Bst II, Indel192, Indel101, Indel752*, and *Indel753* were located on chromosome 6A. The results showed that SN0293-2 and SN0293-7 inherited genetic components from *Th. ponticum*, so they were wheat-*Th. ponticum* introgression lines.

**Table 1:** Markers developed specific to *Thinopyrum ponticum*.

**Markers**	**Homeologous groups**	**Sequences**
*CAPS421-HaeIII*	2	F: 5′-GAACCCCGGATCTGAGTGTCCA-3′ R: 5′-CAACCAACTGCGCTGTCCGTC-3′
*CAPS761-SacI*	6	F: 5′-GAAATATCCAACCAGAACAGTGG-3′ R: 5′-CTCTGCTTGAGTGGCAGGACT-3′
*CAPS564-BstEII*	6	F: 5′-ATCCAAACAAGACAACCCGTCTTG-3′ R: 5′-GCTTGTCTATACCCTAGTCGCGT-3′
*Indel192*	6	F: 5′-ACTCCCAAGGGTGAACCTATGAT-3′ R: 5′-CGGTCAGAGGTAACTTGCTGTG-3′
*Indel101*	6	F: 5′-TAGACCTTCGTGGGAACCTTTG-3′ R: 5′-TAGACCTTCGTGGGAACCTTTG-3′
*Indel752*	6	F: 5′-TACGGCTAAAGGAGTTGACC-3′ R: 5′-TGATGCTGTGGGAACGAAA-3′
*Indel753*	6	F: 5′-AACGCTAAGACTGGATTGATTG-3′ R: 5′-ACCTAATGCGACAGATGGACAA-3′

### Analyses of Agronomic Traits

**Figure 7:**
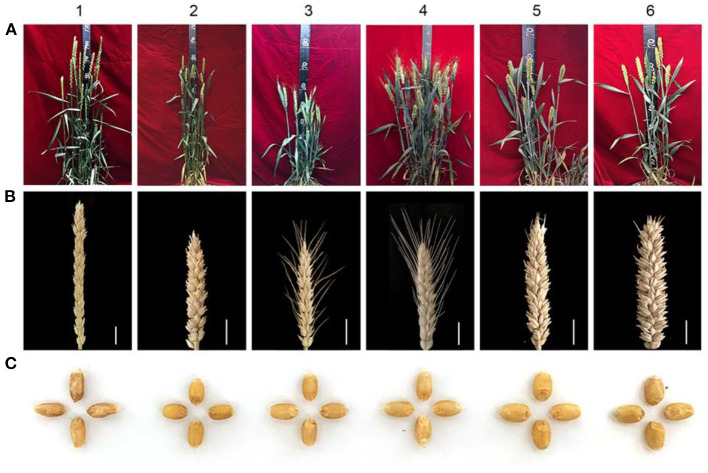
Plants, spikes, and kernels of SN0293-2, SN0293-7, and their parents. **(A)** Plants. **(B)** Spikes. **(C)** Kernels. 1–6 refer to SNTE20, YN15, SN224, JM22, SN0293-2, and SN0293-7, respectively. Scale bar = 2 cm.

The agronomic traits of SN0293-2 and SN0293-7 were compared with those of their parents (Figure 7). SN0293-2 and SN0293-7 displayed average plant heights of 65.4 ± 3.1 cm and 69.3 ± 4.2 cm, respectively, which were lower than the parents SNTE20 (105.2 ± 3.9 cm), YN15 (84.0 ± 2.4 cm) and JM22 (83.5 ± 2.7 cm) except SN224 (61.0 ± 3.6 cm). They shared about equal numbers of spike length and spikelets as the common wheat parents. However, SN0293-2 and SN0293-7 produced more kernels per spike (56 ± 5 and 67 ± 3, respectively) and higher 1,000-grain weight (53.8 ± 1.7 and 53.0 ± 1.3, respectively). These agronomic data indicated that the two introgression lines probably carried genes that were beneficial for wheat breeding.

## Discussion

Wild relatives of wheat have been serving as valuable gene reservoirs due to their resistance to many biotic and abiotic stresses. In the past few decades, wide crosses have been employed to incorporate useful genes into common wheat. The tall grass *Th. ponticum* was extensively used in wheat genetic improvement. The elite cultivars, such as Xiaoyan 6, Gaoyou 503, Xiaoyan 60, and Xiaoyan 81, were widely planted in China and had played an important role in wheat production (Li et al., 2015; Luo et al., 2021; Yang et al., 2022). Meanwhile, numerous chromosome engineering materials were generated, including partial amphiploids (Zheng et al., 2015), additions (Li et al., 2016), substitutions (Wang et al., 2019; Li et al., 2021), translocations (Yang et al., 2022) and introgression lines (Zhan et al., 2014).

In the present study, *Th. ponticum*, SNTE20 and wheat-*Th. ponticum* introgression lines SN0293-2 and SN0293-7 displayed excellent resistance to powdery mildew. Up to now, 11 genes have been officially designated from *Th. ponticum* (Li and Wang, 2009; Liu et al., 2020). Among them, only one gene named *Pm51* was responsible for resistance to powdery mildew. At the seedling stage, the two introgression lines were susceptible to the *Bgt* isolate E20 (Supplementary Table 1), while *Pm51* was nearly immune (Zhan et al., 2014). As for the *Bgt* isolate E09, common wheat parents SNF63, YN15, and SN224 showed susceptibility but JM22 displayed immunity because JM22 carried two resistance genes *Pm2* and *Pm52* (Cao et al., 2010; Qu et al., 2020). Molecular marker analyses demonstrated that both SN0293-2 and SN0293-7 carried these two powdery mildew resistance genes as well. At the adult plant stage, JM22 and CH7086 exhibited susceptibility to powdery mildew similarly to the other common wheat parents (Figure 1 and Supplementary Figure 2), suggesting that *Pm2, Pm51*, and *Pm52* lost resistance in Tai'an, Shandong Province, China. However, resistance of the two introgression lines remained as well as *Th. ponticum* and SNTE20. Seen from the results above, the seedling resistance of SN0293-2 and SN0293-7 originated from either *Th. ponticum* or JM22, and the adult plant resistance of them was putatively derived from *Th. ponticum* obviously different from *Pm51*. Segregating populations are being developed for mapping the adult plant resistance. In addition, 1RS, herein, had no resistance to powdery mildew since the T1BL·1RS translocation SN224 exhibited consistent susceptibility at both the seeding stage and adult plant stage.

GISH and FISH have been used as powerful tools to detect alien chromosomes or chromosome segments and chromosomal structural variations in distant hybridization. In this study, one pair of T1BL·1RS was confirmed by GISH-FISH in SN0293-2, but no cytological evidence for *Th. ponticum* was found in SN0293-2 and SN0293-7, even though some structural variations were demonstrated between these two lines and their parents. The alien segments must be too small to be detected for cytogenetic tools due to the accuracy limitation. Recently, the wheat SNP array has been employed to identify wild relatives and their derivatives with wheat. Zhou et al. (2018) constructed the genetic linkage map of *Agropyron cristatum* and characterized a number of wheat-*A. cristatum* chromosome lines. Several wheat-*Th. ponticum* translocation and introgression lines were also subjected to Wheat 660K SNP array and deletion events were detected (Li et al., 2019; Yang et al., 2021, 2022). In order to determine whether SN0293-2 and SN0293-7 inherited genetic components from *Th. ponticum* or not, the Axiom Wheat 660K Genotyping Array were also used in the present study. One hundred and fifty-seven SNPs were found to be specific to *Th. ponticum*. These SNPs referred to 9 chromosomes of wheat genomes, 134 of which were located on the wheat chromosome 6A (Figure 5). Then three CAPs markers were developed and showed specificity to *Th. ponticum* in SN0293-2 and SN0293-7. Similarly, four Indel markers belonging to homoeologous group 6 were obtained according to the re-sequence data (Table 1 and Figure 6). It was indicated that the wheat SNP array has higher accuracy than cytological methods and could effectively work in identification of wheat-*Th. ponticum* introgression lines.

Most of the alien chromosome lines experience more or less penalties of agronomic traits because of linkage drags of the whole alien chromosomes or large alien segments they carry. For instance, many addition lines generally have longer growth duration and smaller grains due to the introduced alien chromosomes. However, introgression lines have small alien segments that GISH cannot detect, generally resulting in no or less genetic drag. Therefore, germplasms of this type with target genes usually exhibit positive agronomic traits and are considered to be the most valuable materials for wheat breeding. In order to breed disease-resistant lines with favorable agronomic traits, SN224 and JM22 were used as male parents. The former is a breeding line with dwarf stems developed in our lab. The latter is an elite cultivar with the largest promotion area in the last decade in China (Jia et al., 2020). JM22 was also employed as the last parent to improve agronomic traits and to provide two *Pm* genes. Consequently, the wheat-*Th. ponticum* introgression lines SN0293-2 and SN0293-7 carried a new resistance gene putatively from *Th. ponticum* and pyramided *Pm2* and *Pm52* derived from JM22. They also showed positive agronomic traits, such as more kernels and higher 1,000-weight, enhancing the application potential in wheat breeding programs.

## Conclusions

Two wheat-*Th. ponticum* introgression lines were developed and named SN0293-2 and SN0293-7, respectively. Besides *Pm2* and *Pm52*, they possessed a new powdery mildew resistance gene *PmSN0293* putatively from *Th. ponticum* obviously different from *Pm51* reported previously. Seven markers were obtained and confirmed to be specific to *Th. ponticum*. Superior resistance to powdery mildew at both the seedling and adult plant stages and positive agronomic traits give the two introgression lines great potential to be used in wheat breeding programs.

## Data Availability Statement

The data presented in the study are deposited in the Genome Sequence Archive in BIG Data Center repository (https://bigd.big.ac.cn/), accession number PRJCA009783

## Author Contributions

YB designed the research. ML and YY performed the experiments. FN and XL analyzed the data and developed the specific markers. HW contributed to the development of the materials. ML and YB wrote the manuscript. All authors approved the final version of the manuscript.

## Funding

This project was supported by the National Natural Science Foundation of China (No. 32071998), the National Key Research and Development Program of China (No. 2016YFD0102004-02) and Key R&D Program of Shandong Province (Major Science and Technology Innovation Project) (2021LZGC009).

## Conflict of Interest

The authors declare that the research was conducted in the absence of any commercial or financial relationships that could be construed as a potential conflict of interest.

## Publisher's Note

All claims expressed in this article are solely those of the authors and do not necessarily represent those of their affiliated organizations, or those of the publisher, the editors and the reviewers. Any product that may be evaluated in this article, or claim that may be made by its manufacturer, is not guaranteed or endorsed by the publisher.
