# Supplementary figures and images for: Characterization of Two Wheat-Thinopyrum ponticum Introgression Lines With Pyramiding Resistance to Powdery Mildew
Source: Front Plant Sci. 2022 Jul 15;13:943669. doi: 10.3389/fpls.2022.943669 (PMC9335053; doi:10.3389/fpls.2022.943669)

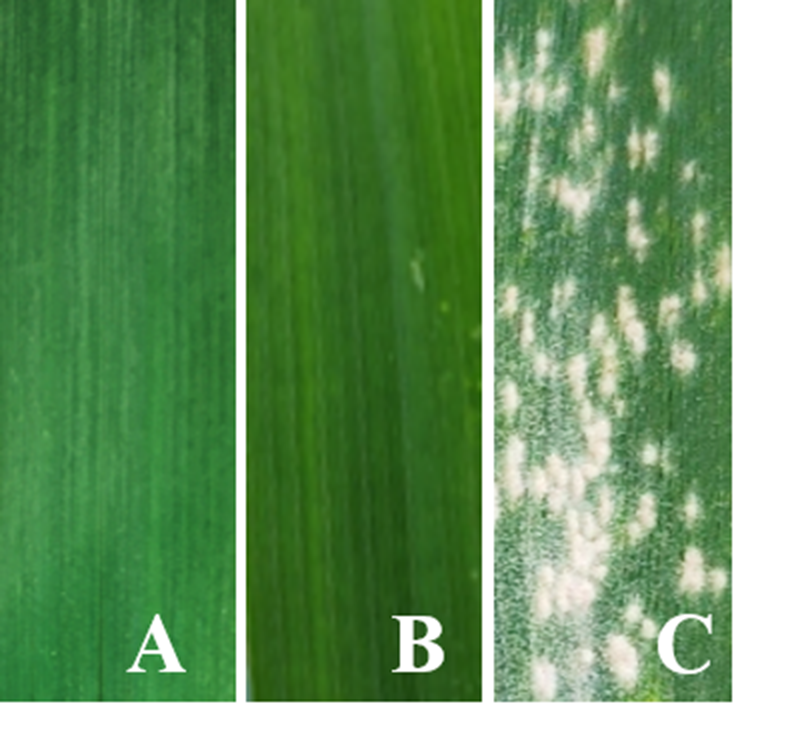

Supplement: Supplementary file 3 [file Image_2.tif]
